# Bone health in juvenile idiopathic arthritis compared with controls based on a Norwegian observational study

**DOI:** 10.1136/rmdopen-2025-005605

**Published:** 2025-06-19

**Authors:** Anette Lundestad, Lena Cetrelli, Oskar Welander Angenete, Thomas Angell Augdal, Karin Tylleskär, Ellen Berit Nordal, Karen Rosendahl, Gry Børmark Hoftun, Mari Hoff, Pål Richard Romundstad, Marite Rygg

**Affiliations:** 1 Department of Clinical and Molecular Medicine (IKOM), Norwegian University of Science and Technology, Trondheim, Norway; 2 Department of Pediatrics, Trondheim University Hospital St Olav’s Hospital, Trondheim, Norway; 3 Center for Oral Health Services and Research, TkMidt, Trondheim, Norway; 4 Department of Circulation and Medical Imaging (ISB), Norwegian University of Science and Technology, Trondheim, Norway; 5 Department of Radiology and Nuclear Medicine, Trondheim University Hospital St Olav’s Hospital, Trondheim, Norway; 6 Section of Paediatric Radiology, University Hospital of North Norway, Tromsø, Norway; 7 Department of Clinical Medicine, UiT The Arctic University of Norway, Tromsø, Norway; 8 Child and Youth Clinic, Haukeland University Hospital, Bergen, Norway; 9 Department of Pediatrics, University Hospital of North Norway, Tromsø, Norway; 10 Department of Neuromedicine and Movement Science (INB), Norwegian University of Science and Technology, Trondheim, Norway; 11 Department of Rheumatology, Trondheim University Hospital St Olav’s Hospital, Trondheim, Norway; 12 Department of Public Health and Nursing (ISM), Norwegian University of Science and Technology, Trondheim, Norway

**Keywords:** Arthritis, Juvenile, Bone Density, Child

## Abstract

**Background:**

Children with juvenile idiopathic arthritis (JIA) are at risk for impaired bone health. This study evaluates bone mineral density (BMD) and potential risk factors for reduced BMD.

**Methods:**

In the NorJIA study, Norwegian children with JIA, and age-matched and sex-matched controls participated in a multicentre cohort study with clinical examinations, questionnaires, imaging and blood tests. BMD was measured using dual-energy X-ray absorptiometry and adjusted for bone age. Standard descriptive statistics and t-tests were used.

**Results:**

205 children with JIA had BMD measured at two study visits, 2 years apart and 125 controls at the second visit. At visit 2, median age was 14.7 years (IQR 11.5–16.6). Median disease duration was 6.6 (IQR 4.7–10.4) years, 50.7% had used or were currently using biologic disease-modifying antirheumatic drugs and 25.9% had ever used systemic steroids. There were no substantial differences in BMD Z-scores between the JIA group and controls. Mean BMD Z-score L1-L4 in JIA was 0.0 (95% CI −0.1, 0.1) and in controls 0.1 (95% CI −0.1, 0.3). A robust association was seen between physical activity levels and BMD. In children with JIA, the mean BMD Z-score L1-L4 was −0.3 (95% CI −0.6, 0.0) in the low-activity group and 0.2 (95% CI 0.0, 0.4) in the high-activity group, with a similar trend in controls. Children with JIA were as physically active as controls.

**Conclusions:**

BMD Z-scores in JIA were similar to controls and positively associated with physical activity. This underlines the importance of early disease control, steroid-sparing medications and physical activity to optimise bone health.

**Trial registration number:**

NCT03904459.

**WHAT IS ALREADY KNOWN ON THIS TOPIC**

- Previous data indicate that children with juvenile idiopathic arthritis (JIA) have decreased bone mineral density (BMD) and are less active, when compared with controls.
- Little is known about bone health in children with JIA treated in the era of biologic treatment.

**WHAT THIS STUDY ADDS**

- This study did not find any significant differences in BMD Z-scores between children with JIA and controls.
- We found lower BMD Z-scores associated with reduced physical activity levels in both study groups.
- Both groups demonstrated similar levels of physical activity.

**HOW THIS STUDY MIGHT AFFECT RESEARCH, PRACTICE OR POLICY**

- Physical activity, together with early disease control and use of steroid-sparing medications, should be part of the recommendations to optimise bone health in children with JIA.

## Background

 Juvenile idiopathic arthritis (JIA) is the most common rheumatic disease in childhood with an onset before the age of 16 years. It is a heterogeneous condition with ongoing arthritis of unknown aetiology, persisting for at least 6 weeks where other known conditions are excluded.^1^ A high variation in incidence and prevalence rates between countries is seen.^2^ The Nordic countries generally have a high incidence rate, with 25.9 per 100 000 children per year in northern and 17.9 in central Norway.^3^ Diagnostics, treatment options and overall outcome have improved after the goal and strategy changed to treat-to-target clinically inactive disease and remission, by early use of disease-modifying anti-inflammatory drugs (DMARDs), including biological DMARDs (bDMARDs). As new drugs have decreased the use of prolonged glucocorticoids, long-term outcomes such as pubertal delay, growth suppression and physical disability have improved.^4^

Bone mineral density (BMD) in adulthood largely depends on the peak bone mass achieved during childhood and adolescence and the subsequent gradual bone loss. Over 35% of the total amount of bone mineral in adulthood has been reported to be gained during the 4-year adolescent phase that coincides with peak linear growth velocity.^5^ A high peak bone mass, typically reached at 20–25 years of age, contributes to a larger reserve later in life and positively influences the fracture risk.^6^ It is therefore important to optimise bone gain in these valuable years and focus on factors known to play a role in bone mass accrual, such as weight, physical activity, calcium, vitamin D and protein intake.^7^ Dual-energy X-ray absorptiometry (DXA) is the gold standard for assessing BMD due to its safety, low cost, speed and precision.^8 9^ The International Society for Clinical Densitometry (ISCD) Paediatric Positions recommend assessing bone health in paediatric patients primarily at the lumbar spine (LS) and the total body less head (TBLH).^8^ However, other skeletal sites may be considered if these primary sites are not practical.^10^ Digital X-ray radiogrammetry (DXR) is a promising method for assessing bone health in children. Among other bone imaging techniques, DXR has demonstrated the most significant positive correlation with DXA. Further research is needed to confirm DXR’s reliability in evaluating bone health and predicting fractures in children and adolescents.^9^ Children with JIA are at higher risk of decreased BMD compared with healthy children due to several different factors, such as genetics, chronic inflammation, medication side effects, delayed puberty, inadequate nutrition and low physical activity.^4 11^ It is crucial to identify individuals at risk of reduced bone mass early in the disease process.^12^ Knowledge about the factors influencing bone health and long-term outcomes in children with JIA during the era of biological treatments remains limited. The aim of this study was to examine bone health in a group of Norwegian children and adolescents with JIA and controls, and to identify risk factors for reduced bone health to facilitate intervention strategies.

## Methods

### Study design

The NorJIA study (www.norjia.com) is a Norwegian, prospective, multicentre clinical cohort study, enrolling participants between March 2015 and November 2020. Children and adolescents with JIA were recruited from outpatient Paediatric Rheumatology clinics at St. Olavs University Hospital in Trondheim, Haukeland University Hospital in Bergen and University Hospital of North Norway in Tromsø. All children with JIA aged 4–16 years who were consecutively seen at the three centres during the study period were invited to participate. Included children performed two study visits, the first visit with random disease duration, and the second visit after 24 months (±3 months). Age-matched and sex-matched controls were recruited from both rural and urban areas and were organised by the Center of Oral Health Services and Research (TkMidt) in Trondheim, the Public Dental Health Service Competence Centre of Northern Norway (TkNN) in Tromsø and the Oral Health Centre of Expertise in Western Norway (TkV) in Bergen. The controls were invited to participate during their scheduled regular oral health examinations, which are part of the free national healthcare programme mandated by the Norwegian Directorate of Health. Inclusion criteria for participation in the NorJIA study were a diagnosis of JIA according to The International League of Associations for Rheumatology (ILAR) criteria, age between 4 and 16 years, together with a signed informed consent. Age-matched and sex-matched controls were eligible if they did not have JIA or other rheumatic conditions. For this particular study on bone health, we included all participants with JIA and a DXA scan of the LS (L1-L4) performed at both study visits, and controls with a DXA scan performed at visit 2, the only time BMD was measured in controls.

### Data collection

Experienced paediatric rheumatologists examined the children and adolescents with JIA at each of the three study centres. Demographic, anthropometric, clinical and imaging data were registered from both study visits, including a radiograph of the left hand and a DXA scan. Data from patient-reported questionnaires and blood tests were also collected. For controls, anthropometric data and information from self-reported questionnaires were collected. At the second visit, controls from two of the study centres (Tromsø and Trondheim) also underwent a DXA scan, a radiograph of the left hand and blood tests.

### Measures

#### Sociodemographics and anthropometrics

Sex, age, height, weight and parenteral education level were registered. The highest level of education for both parents was used and reclassified into three levels: Low level (≤ 13 years), intermediate (university education up to 4 years) and high (university education ≥5 years). Body mass index (BMI) was calculated (weight (kg))/(height (metres(m))^2^) and adjusted for age and sex to get iso-BMI groups, corresponding to adult BMI groups, including underweight ≤18.5 kg/m^2^, and overweight/obesity ≥25 kg/m^2^, according to The International Obesity Task Force recommendations.^13^

#### Vitamin D and calcium intake

Vitamin D and calcium intake were estimated using a self-reported Food Frequency Questionnaire (FFQ). Parents or legal guardians completed the FFQ for children under 12 years, while those aged 12 and older completed it themselves. Mean daily intake (µg/day for vitamin D and mg/day for calcium) from food and supplements was calculated separately and then combined. Inadequate intake was categorised as <10 µg/day for vitamin D, <800 mg/day (ages 4–10 years) and <1150 mg/day (ages 11–18 years) for calcium, based on recommendations from the Norwegian Directorate of Health. An experienced clinical nutritionist conducted the nutritional assessments.

#### Health behaviour

The questions on high-intensity physical activity were adopted from the WHO Health Behavior in Schoolchildren (WHO HBSC); ‘Not during the average school day: How many days a week do you play sports or exercise to the point where you breathe heavily and/or sweat?’ The six response alternatives were collapsed into three levels; 4 days a week or more, 2–3 days a week and 1 day a week or less, allowing us to divide the participants into high, moderate and low activity levels, as in previous studies.^14 15^ For children under 12 years of age, parents or legal guardians assisted the child in completing the questionnaire or answered the questions on their behalf.

#### Disease characteristics and outcome

JIA-specific clinical characteristics included age at disease onset, disease duration, JIA category according to the ILAR classification criteria,^1^ disease activity, medication and results of blood tests including human leucocyte antigen B27 (HLA-B27). Anti-nuclear antibodies (ANA) and rheumatoid factor (RF) were measured around the time of diagnosis and tested twice, at least 3 months apart. ANA was measured with indirect immunofluorescence with human epithelial cells (HEp-2 cells).

Disease status and activity were assessed with the Wallace and the American College of Rheumatology (2004/2011) provisional criteria,^16 17^ in addition to the physician’s global assessment of disease activity scored on a 21-numbered circle Visual Analogue Scale (PhysGA VAS) (0=no disease activity, 10=highest disease activity). Remission off medication was defined as inactive disease for ≥12 months. Inactive disease was defined as no joints with active arthritis, no rash, fever, serositis, splenomegaly or generalised lymphadenopathy secondary to JIA, no active uveitis, normal erythrocyte sedimentation rate (ESR) <20 mm/hour or C reactive protein (CRP) <10 mg/L if taken, PhysGA VAS=0 and morning stiffness ≤15 min. Active disease was defined as either a flare or continuous disease since onset. Flare was defined as one or more episodes of clinically significant worsening, with a change from inactive to active disease requiring a medication adjustment. Continuous disease was referred to persistently active disease without achieving inactive disease or remission. Disease activity was also assessed and quantified by the Juvenile Arthritis Disease Activity score based on 71 joints (JADAS71).^18^ JADAS71 was calculated based on the PhysGA (range 0–10), the patient/parent global assessment of disease impact on well-being (PatGA) scored on a 21-numbered circle VAS (0=no impact, 10=maximal impact),^19^ the number of active joints (range 0–71) and the ESR (CRP if missing ESR) normalised to 0–10, total score 0–101 (0=no activity, 101=highest disease activity). Children with JIA (≥9 years) or parent/legal guardian’s (for children <9 years) reported functional ability with the Childhood Health Assessment Questionnaire, total score 0–3 (0=no disability, 3=maximal disability)^20^ and PatGA.^19^

#### Treatment

For the children with JIA, the past and/or current use and type of medication was registered at both visits and divided into three groups: Never used any DMARDs, only used conventional synthetic DMARDs, including methotrexate, ciclosporin, mycophenolate mofetil, hydroxychloroquine or ever used bDMARDs including etanercept, infliximab, adalimumab, tocilizumab, abatacept, rituximab and canakinumab. No children had used targeted synthetic DMARDs. The children were also subgrouped into ever or never used systemic steroids.

#### Blood samples

A standardised set of blood samples, including serum 25-OH-Vitamin D (nmol/L) and ionized calcium (mmol/L), was collected at both visits from the children with JIA and at visit 2 from controls. The study visits were performed throughout the year from August to June.

### DXA measurements

BMD was measured using DXA, Lunar iDXA in Bergen and Trondheim, and Lunar Prodigy in Tromsø (GE Lunar Corporation, Madison, Wisconsin, USA). BMD from the LS L1-L4, femoral neck, total hip, TBLH, and body composition with total fat percentage were measured in all study participants in accordance with the ISCD Paediatric Official Position.^8^ Participants with orthopaedic implants (n=2) did not perform DXA of the affected site. BMD (in g/cm^2^) was calculated using the paediatric software with an integrated reference database and used to estimate the BMD Z-scores, defined as the number of SDs above or below the mean value expected for age and sex. The database includes BMD data from the general population of the USA, previously validated as suitable for clinical use in the paediatric Norwegian population.^21^ There was no change in hardware during the study period, but the software was upgraded once in Trondheim. Since one cannot directly obtain bone age-adjusted (BA-adjusted) BMD Z-scores using the GE software, we performed this task manually using the BA data from BoneXpert (see below), so that new calculations could be made.

### BA assessment and Bone Health Index

For participants with JIA, a radiograph of the left hand as per protocol was performed at both visits, while controls residing in Trondheim or Tromsø had one radiograph taken at the second visit. From the hand radiographs, BA and Bone Health Index (BHI) were analysed and calculated using the manufacturers software (BoneXpert Standalone V.3.2.1.12 Visiana, Holte, Denmark), a method validated on a variety of ethnicities and patients groups,^22^ previously described by Thodberg *et al*.^23^ The BA is automatically measured, assessing a skeletal age from the average of 21 tubular bones in a digitalised radiograph of the left hand with a three-layer image analysis.^23^ BHI SD score (BHI-SDS) can be used to compare the observed BHI with the BHI of the general population of the same BA and gender (ie, the SD divided by the mean). BHI-SDS is derived from the automated measurements of metacarpal width, length and cortical thickness.

### Statistical analysis

Descriptive statistics were used for demographic, anthropometric and clinical characteristics of JIA and controls and disease-related characteristics of the JIA group. Categorical data are presented as frequency and percentages, while continuous variables are presented as median and IQR or mean and SD. The BMD L1-L4 Z-score and BA-adjusted BMD L1-L4 Z-score results are presented as mean with 95% CIs. Total fat is presented as a percentage with 95% CI. A paired t-test was used to compare the difference in mean BMD Z-scores, BHI-SDS and total fat for children with JIA between visits 1 and 2 and to estimate the 95% CI. A two-sample t-test was used to compare the difference in mean BMD Z-scores, BHI-SDS and total fat between JIA and controls at visit 2. Data were analysed using STATA V.18.0 software (StataCorp. 2023. Stata Statistical Software: Release 18, StataCorp).

## Results

**Figure 1:**
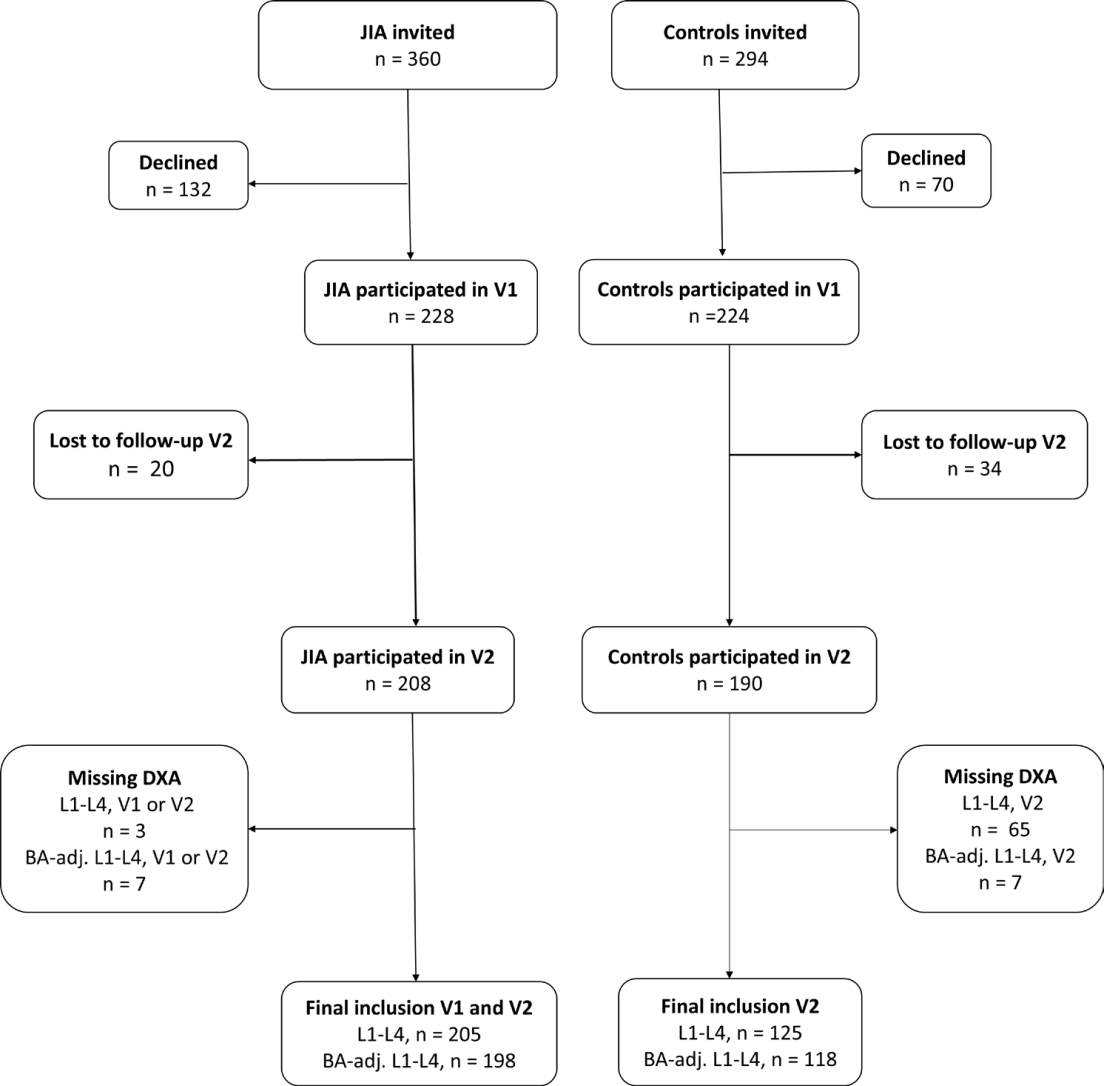
Flow chart of participating children and adolescents with juvenile idiopathic arthritis (JIA) and controls. (BA automatically measured by the software program BoneXpert, which assesses skeletal age from 21 bones in a digitalised radiograph of the left hand). BA-adj., bone age-adjusted; DXA, dual-energy X-ray absorptiometry; L1-L4, lumbar spine from L1 to L4; V1, visit 1; V2, visit 2.

Of the 360 children with JIA who were invited to participate in the NorJIA study, 228 accepted and participated at visit 1 (figure 1). After 2 years (±3 months), 11 boys and 9 girls were lost to follow-up or did not consent to further participation. Of the 208 children with JIA and two study visits in the NorJIA cohort, three were missing data on BMD L1-L4, leaving 205 children with JIA in the final study population. Of these, 7 did not have available data for BA estimates, resulting in 198 participants with BA-adjusted L1-L4 (BA-adj. L1-L4) at both visits. In total, 125 controls from Trondheim and Tromsø had available blood tests and DXA with BMD L1-L4 at visit 2, including 118 with BA-adj. L1-L4. The median age of children with JIA who declined to participate was 10.7 years, 2 years younger than those who participated.

### Sociodemographic and health characteristics

Of the 205 children with JIA, 60% were female, and median age at visit 2 was 14.7 years; similar figures were observed in the control group (table 1). No substantial difference was seen in parental education and Iso-BMI, although there was a trend towards a slightly higher proportion of parents with higher education in the control group (37.2% vs 35.6%). The proportion of children with overweight/obesity was slightly higher in the JIA group compared with controls (22.6% vs 16.8%). Serum levels of vitamin D, ionised calcium and physical activity were comparable in the two groups. Supplemental intake of both vitamin D and calcium was significantly higher in children with JIA compared with controls. A slightly higher proportion of controls had inadequate intake.

**Table 1:** Characteristics of the study groups

Visit 2	JIA	Controls
Total, n	205	125
Female, n (%)	123 (60.0)	79 (63.2)
Age at visit, years, median (IQR)	14.7 (11.5–16.6)	14.6 (11.3–16.7)
Parental education*, n (%)		
High	72 (35.6)	32 (37.2)
Intermediate	72 (35.6)	34 (39.5)
Low	58 (28.7)	20 (23.3)
Iso-BMI†, n (%)		
Underweight	10 (4.9)	4 (3.2)
Normal	148 (72.6)	100 (80.0)
Overweight/obese	46 (22.6)	21 (16.8)
Serum 25-OH-vitamin D‡ (nmol/L)		
Mean (SD)	55.1 (20.5)	55.4 (20.2)
≤50, n (%)	77 (37.9)	46 (42.2)
Daily vitamin D intake§		
μg/day, mean (SD)	7.9 (8.5)	8.6 (9.1)
Supplemental intake, n (%)	131 (69.7)	30 (28.8)
Inadequate intake, n (%)	146 (77.7)	71 (68.3)
Ionised calcium¶ (mmol/L)		
Mean (SD)	1.24 (0.0)	1.25 (0.0)
≤1.22, n (%)	43 (22.1)	14 (12.7)
Daily calcium intake**		
mg/day, mean (SD)	806.6 (530.2)	683.1 (498.6)
Supplemental intake, n (%)	19 (10.1)	4 (3.6)
Inadequate intake, n (%)	133 (70.7)	91 (82.0)
Physical activity††, n (%)		
High	72 (38.5)	40 (37.0)
Intermediate	69 (36.9)	44 (40.7)
Low	46 (24.6)	24 (22.2)

* Parental education: Low level is defined as both parents with education ≤13 years, Intermediate: One or both parents with university education up to 4 years, High: One or both parents with university education 5 years or more. JIA, missing 3. Controls, missing 39.

† Iso-BMI according to the International Obesity Task Force recommendations corresponding to adult BMI (underweight: BMI≤18.5 kg/m2, overweight: BMI 25–29.9, obesity BMI≥30), overweight and obesity combined. JIA, missing 1.

‡ Sampled once, at study visit 2, taken place throughout the year, August–June. Deficiency=≤50 nmol/L. JIA, missing 2. Controls, missing 16.

§ Estimates (dietary and supplemental) based on data from self-reported FFQ. JIA, missing 17. Controls, missing 21. Inadequate intake defined as ≤10 µg/day, according to national recommendations.

¶ Sampled once, at study visit 2. JIA, missing 10. Controls, missing 15. Deficiency defined as ≤1.22 nmol/L.

** Estimates (dietary and supplemental) based on data from self-reported FFQ. JIA, missing 17. Controls, missing 14. Inadequate intake defined as ≤800 mg/day (age 4–10 years), ≤1150 mg (age 11–18 years), according to national recommendations. (Supplemental intake). JIA, missing, 14. Controls, missing 14.

†† Physical activity question adopted from the WHO Health Behaviour in Schoolchildren: ‘Not during the average school day, how many days per week do you play sports or exercise to the point where you breathe heavily and/or sweat?’ The responses were divided into three categories. High activity: ≥4 times per week, intermediate activity: 2–3 times per week, low activity: 1 time per week or less. JIA, missing 18. Controls, missing 17.

BMI, body mass index; FFQ, food frequency questionnaires; JIA, juvenile idiopathic arthritis.;

### Disease characteristics

The median age at disease onset was 6.1 years, and median disease duration at visit 2 was 6.6 years (table 2). All JIA categories were represented, and oligoarticular persistent JIA was the largest group (31.2%). The disease status changed slightly from visit 1 to visit 2, with more children with inactive disease (44.4%) and slightly fewer children with active disease (35.6%) at visit 2. The number of children using bDMARDs increased by 10.2 percentage points (95% CI 0.7, 19.8) from 40.5% at visit 1 to 50.7% at visit 2, while the proportion of patients that had ever used systemic steroids was 25.9% at visit 2.

**Table 2:** Disease characteristics of children in the JIA study group, n=205

Disease characteristics	Values
Age at disease onset, years, median (IQR)	6.1 (2.2–10.3)
ANA positive*, n (%)	66 (32.2)
RF positive, n (%)	4 (2.0)
HLA-B27 positive, n (%)	59 (28.8)

	Visit 1	Visit 2
Disease duration, years, median (IQR)	4.6 (2.7–8.4)	6.6 (4.7–10.4)
Age at visit, years, median (IQR)	12.7 (9.6–14.7)	14.7 (11.5–16.6)
JIA category†, n (%)		
Oligoarticular persistent	69 (33.6)	64 (31.2)
Oligoarticular extended	22 (10.7)	25 (12.2)
Polyarticular RF negative	47 (22.9)	45 (22.0)
Polyarticular RF positive	3 (1.5)	3 (1.5)
Psoriatic arthritis	8 (3.9)	10 (4.9)
Enthesitis-related arthritis	22 (10.7)	24 (11.7)
Systemic arthritis	7 (3.4)	7 (3.4)
Undifferentiated arthritis	27 (13.2)	27 (13.2)
DMARDs ever‡, n (%)		
No DMARDS	45 (22.0)	36 (17.6)
csDMARDS only	77 (37.6)	65 (31.7)
bDMARDS	83 (40.5)	104 (50.7)
Systemic steroids ever, n (%)		
No systemic steroids	159 (77.6)	152 (74.2)
Systemic steroids ever	46 (22.4)	53 (25.9)
Disease status§, n (%)		
Remission off medication	54 (26.3)	41 (20.0)
Inactive disease	68 (33.2)	91 (44.4)
Active disease	83 (40.5)	73 (35.6)
JADAS71¶, mean (SD)	3.0 (3.6)	2.6 (3.0)
PhysGA**>0, n (%)	73 (35.6)	54 (27.0)
PatGA††>0, n (%)	145 (72.5)	128 (66.0)
CHAQ‡‡>0, n (%)	114 (57.3)	95 (49.0)

* Number of children with two available ANA tests, missing 2.

† JIA category according to The International League of Associations for Rheumatology classification criteria.

‡ csDMARDs include methotrexate, ciclosporin, mycophenolate mofetil, hydroxychloroquine. bDMARDs includes etanercept, infliximab, adalimumab, tocilizumab, abatacept, rituximab, canakinumab.

§ Disease status according to Wallace *et al*^17^ and Wallace *et al*.^16^ Remission off medication=inactive disease off medication for ≥12 months. Inactive disease=inactive disease off medication for less than 12 months, or inactive disease on medication independent of the duration of the inactive state. Active disease=flare or continuous active disease.

¶ JADAS71 includes PhysGA (0–10), PatGA (0–10), active joint count 71 joints (0–71) and ESR (normalised to 0–10), score 0–101 (0=lowest, 101=highest). Visit 1, missing 5. Visit 2, missing 15.

** PhysGA, measured on a 21-numbered circle VAS, 0–10 cm (0=no disease activity, 10=highest disease activity). Visit 2, missing 5.

†† PatGA, measured on a 21-numbered circle VAS, 0–10 cm (0=no impact, 10=maximal impact). Visit 1, missing 5. Visit 2, missing 11.

‡‡ CHAQ, total score 0–3 (0=no physical disability, 3=maximal disability). Visit 1, missing 6. Visit 2, missing 11.

ANA, antinuclear antibodies; bDMARDs, biological DMARDs; CHAQ, Childhood Health Assessment Questionnaire; csDMARDs, conventional synthetic DMARDs; DMARDs, disease-modifying antirheumatic drugs; ESR, erythrocyte sedimentation rate; HLA-B27, human leucocyte antigen B27; JADAS71, juvenile arthritis disease activity score based on 71 joints; JIA, juvenile idiopathic arthritis; PatGA, patient’s overall assessment of disease impact on well-being; PhysGA, physician’s global assessment of disease activity; RF, rheumatoid factor; VAS, Visual Analogue Scale.

### Bone mineral density

**Figure 2:**
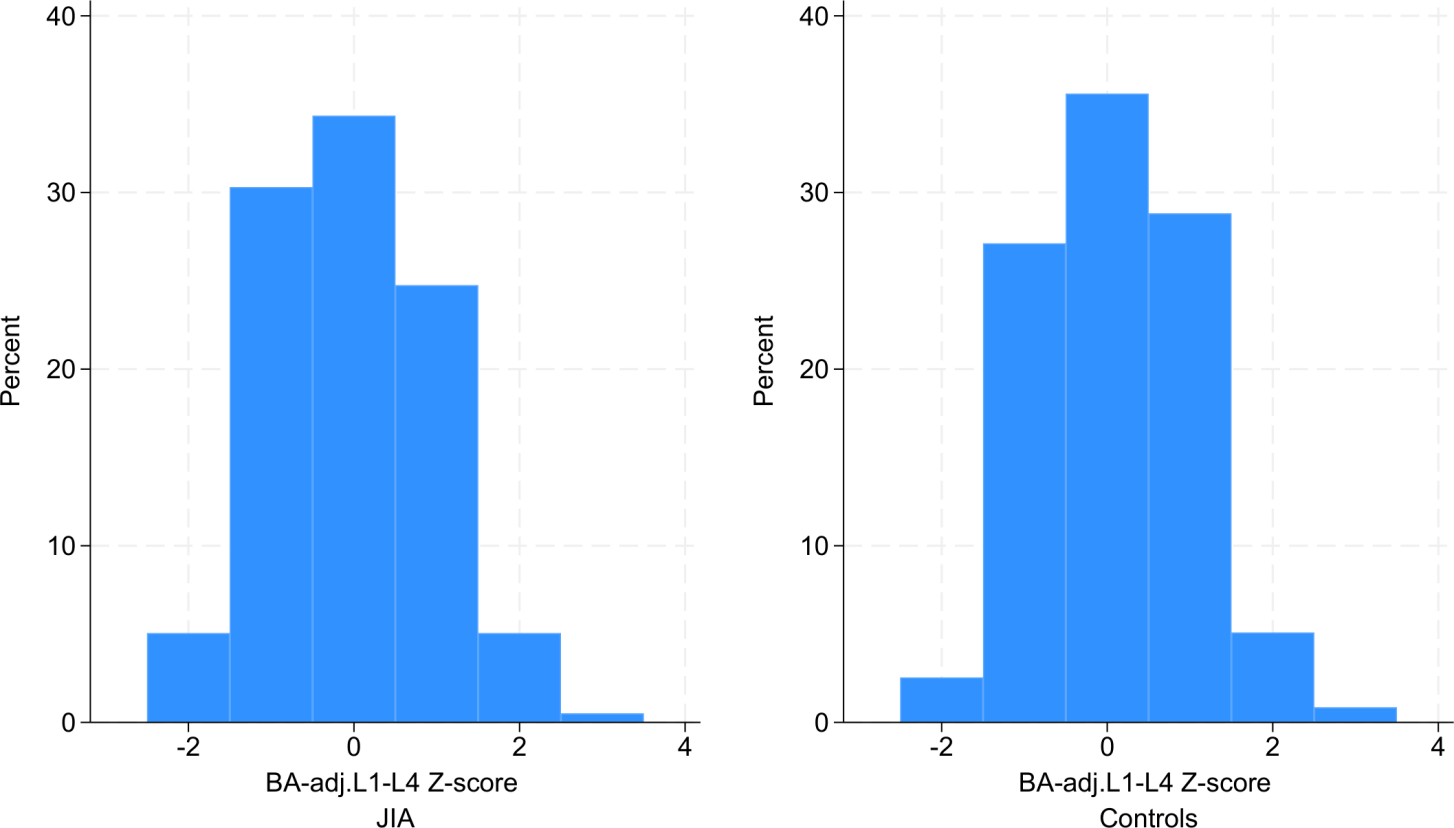
Distribution of lumbar L1-L4 bone age adjusted (BA-adj) bone mineral density (BMD) Z-scores in children with juvenile idiopathic arthritis (JIA) (left) compared with controls (right) at study visit 2. The x-axis of the histogram represents Z-scores, and the y-axis shows the percentage of individuals within each Z-score range. BA was automatically measured by the software program BoneXpert, which assesses skeletal age from 21 bones in a digitalised radiograph of the left hand and used for adjustments of BMD Z-scores.

On average, children with JIA and controls displayed BMD values close to the expected figures with Z-scores close to 0 or slightly above (table 3). Compared with controls, children with JIA tended to have slightly lower BMD Z-scores in all measured locations, and the trend was the same when using BA adjusted BMD Z-scores. However, the differences were minor, and no substantial difference could be found (table 4). In the JIA group, no substantial difference in BMD Z-scores was seen between visit 1 and visit 2, except for TBLH where there was a slight increase with a BA-adjusted mean difference of 0.3 (95% CI 0.2, 0.4) (table 4). When comparing the distribution of all the lumbar BMD Z-scores in the JIA group to the control group at visit 2, most individuals in both groups had Z-scores close to 0. While the control group’s distribution is somewhat more symmetric, the JIA group’s distribution is slightly skewed toward lower BMD Z-scores (figure 2).

**Table 3:** Bone health characteristics and body composition in children with JIA and controls

	JIA				Controls	
	Visit 1		Visit 2		Visit 2	
	Mean(95% CI)N=205	BA-adj. mean(95% CI)N=198	Mean(95% CI)N=205	BA-adj. mean(95% CI)N=198	Mean(95% CI)N=125	BA-adj. mean(95% CI)N=118
BMD Z-score (DXA)						
L1-L4*	0.0 (−0.1, 0.2)	0.1 (−0.1, 0.2)	0.0 (−0.2, 0.1)	0.0 (−0.1, 0.1)	0.1 (0.0, 0.3)	0.1 (−0.1, 0.3)
Left femoral neck†	0.1 (−0.1, 0.2)	0.1 (−0.1, 0.3)	0.1 (0.0, 0.3)	0.2 (0.0, 0.3)	0.3 (0.1, 0.5)	0.3 (0.1, 0.5)
Left hip total†	0.3 (0.1, 0.4)	0.3 (0.1, 0.4)	0.2 (0.1, 0.4)	0.3 (0.1, 0.4)	0.4 (0.2, 0.6)	0.4 (0.2, 0.6)
TBLH‡	−0.1 (−0.2, 0.0)	0.0 (−0.1, 0.1)	0.2 (0.0, 0.3)	0.3 (0.1, 0.4)	0.2 (0.0, 0.4)	0.2 (0.1, 0.4)
BHI–SDS§	−0.5 (−0.7, −0.4)	−0.5 (−0.7, −0.4)	−0.4 (−0.6, −0.3)	−0.4 (−0.6, −0.3)	−0.2 (−0.4, 0.0)	−0.2 (−0.4, 0.0)
Total fat %¶	29.3 (28.1, 30.6)	29.3 (28.1, 30.6)	29.8 (28.6, 31.0)	29.7 (28.5, 31.0)	28.9 (27.4, 30.4)	28.9 (27.4, 30.5)

BA-adj.: BA automatically measured by the software program BoneXpert, which assesses skeletal age from 21 bones in a digitalised X-ray of the left hand.

BMD Z-score: The SD difference between the patient’s BMD measurement assessed with DXA, and the mean BMD of an age-matched and gender-matched reference population.

* BA-adj. JIA, missing 7. BA-adj. controls, missing 7.

† Visit 1; JIA, missing 3. BA-adj. JIA, missing 9. Visit 2; BA-adj. JIA, missing 7. BA-adj. controls, missing 7.

‡ Visit 1; JIA, missing 1. BA-adj. JIA, missing 12. Visit 2; BA-adj. JIA, missing 12. Controls, missing 2. BA-adj. controls, missing 9.

§ Visit 1; JIA, missing 1. BA-adj. JIA, missing 7. Visit 2; JIA, missing 1. BA-adj. JIA, missing 8. Controls, missing 7. BA-adj. controls, missing 8.

¶ Visit 1; BA-adj. JIA, missing 7. Visit 2; BA-adj. JIA, missing 7. Controls, missing 1. BA-adj. controls, missing 8.

BA-adj., bone age-adjusted; BHI-SDS, Bone Health Index SD score; hand X-ray analysed by DXR; BMD, bone mineral density; DXA, dual-energy X-ray absorptiometry; DXR, digital X-ray radiogrammetry; JIA, juvenile idiopathic arthritis; L1-L4, lumbar spine from L1 to L4; TBLH, total body less head.

**Table 4:** Difference in bone health in children with JIA between visits and compared with controls

	Visit 2 vs visit 1		JIA vs control	
	JIA			
	Mean diff.(95% CI)N=205	BA-adj. mean diff.(95% CI)N=198	Mean diff.(95% CI)N=205	BA-adj. mean diff.(CI 95%)N=198
BMD Z-score				
L1-L4*	0.0 (−0.1, 0.0)	−0.1 (−0.1, 0.0)	−0.1 (−0.4, 0.1)	−0.1 (−0.3, 0.1)
Left femoral neck†	0.0 (−0.1, 0.1)	0.1 (0.0, 0.1)	−0.2 (−0.4, 0.1)	−0.1 (−0.4, 0.1)
Left hip total†	0.0 (−0.1, 0.0)	0.0 (−0.1, 0.1)	−0.2 (−0.4, 0.1)	−0.1 (−0.4, 0.1)
TBLH‡	0.3 (0.2, 0.3)	0.3 (0.2, 0.4)	−0.1 (−0.3, 0.2)	0.0 (−0.2, 0.2)
BHI–SDS§	0.1 (0.1, 0.2)	0.1 (0.0, 0.2)	−0.2 (−0.5, 0.0)	−0.2 (−0.5, 0.0)
Total fat %¶	0.5 (−0.1, 1.1)	0.4 (−0.2, 1.1)	0.9 (−1.0, 2.9)	0.8 (−1.2, 2.8)

BA-adj.: BA automatically measured by the software program BoneXpert, which assesses skeletal age from 21 bones in a digitalised X-ray of the left hand.

BMD Z-score: The SD difference between the patient’s BMD measurement assessed with DXA and the mean BMD of an age-matched and gender-matched reference population.

* Visit 1 and 2; BA-adj. JIA, missing 7. BA-adj. controls, missing 7.

† Visit 1; JIA, missing 3. BA-adj. JIA, missing 9. Visit 2; BA-adj. JIA, missing 7. BA-adj. controls, missing 7.

‡ Visit 1; JIA, missing 1. BA-adj. JIA, missing 12. Visit 2; BA-adj. JIA, missing 12. Controls, missing 2. BA-adj. controls, missing 9.

§ Visit 1; JIA, missing 1. BA- adj., missing 7. Visit 2; JIA, missing 1. BA- adj. JIA, missing 8. BA-adj. controls, missing 8.

¶ Visit 1; BA-adj. JIA, missing 7. Visit 2; Controls, missing 1. BA-adj. controls, missing 8.

BA, bone age; BHI-SDS, bone health index SD score; hand X-ray analysed by DXR; BMD, bone mineral density; diff., difference; DXA, dual-energy X-ray absorptiometry; DXR, digital X-ray radiogrammetry; JIA, juvenile idiopathic arthritis; L1-L4, lumbar spine from L1 to L4; TBLH, total body less head.

### Bone Health Index

Using BHI-SDS based on the hand radiograph as another measure of bone health, we observed the same pattern. There was a tendency of a minor difference between the JIA group and controls with a BA-adjusted mean difference of –0.2 (–0.5,0.0), but no substantial difference between visit 1 and visit 2 in children with JIA (table 4).

When comparing individual BHI-SDS values with BA-adjusted BMD L1-L4 Z-scores, the Bland-Altman plot (online supplemental figure 1) indicates no systematic pattern with increasing BMD values, and a slightly lower level measured by the BHI-SDS method. In general, the differences between the measured values according to the two methods fall within the 95% limits of agreement (−2.03, 2.73).

### BMD according to sociodemographic factors

Children with JIA whose parents had low levels of education had the lowest mean BA-adjusted BMD L1−L4 Z-score of −0.3 (95% CI −0.5, –0.1), however, similar results were not seen in the control group (table 5). BMD Z-scores were slightly lower for underweight children compared with normal or overweight children in both children with JIA and controls. Children with JIA with low serum vitamin D or ionised calcium levels tended to have slightly lower BMD compared with those with high levels, but the differences were minor and less apparent among controls. Children with JIA aged 11–18 years with inadequate calcium intake (<1150 mg/day) had slightly lower BMD compared with those aged 4–10 years with inadequate calcium intake (<800 mg/day), with a similar trend observed among controls. A more robust association was seen between physical activity levels and BMD (table 5). The BA-adjusted BMD Z-score in children with low versus high physical activity levels with JIA was −0.3 (95% CI −0.6, 0.0) compared with a Z-score of 0.2 (95% CI 0.0, 0.4). A similar association was observed among the controls. Children with JIA reported physical activity levels comparable to those of the control group.

**Table 5:** BMD and total body fat in children with JIA and controls according to sociodemographic and lifestyle factors

Visit 2	N	JIA (n=198)		N	Controls (n=118)	
		BA-adj. BMD L1-L4, Z-scoreMean (95% CI)	Total fat% (95% CI)		BA-adj. BMD L1-L4, Z-scoreMean (95% CI)	Total fat*% (95% CI)
Parental education†						
High	71	0.1 (−0.1, 0.3)	27.6 (25.8, 29.3)	28	0.0 (−0.4, 0.3)	27.4 (24.0, 30.7)
Intermediate	68	0.2 (0.0, 0.4)	29.8 (27.5, 32.0)	31	0.1 (−0.3, 0.4)	30.1 (27.2, 33.0)
Low	56	−0.3 (−0.5, −0.1)	31.8 (29.3, 34.4)	20	0.3 (−0.2, 0.7)	32.3 (28.1, 36.5)
Iso-BMI‡						
Underweight	10	−0.9 (−1.3, −0.6)	21.5 (16.5, 26.4)	4	−0.4 (−2.8, 2.0)	19.7 (9.3, 30.1)
Normal	143	0.0 (−0.1, 0.2)	27.2 (26.0, 28.4)	93	0.1 (0.0, 0.3)	27.0* (25.6, 28.5)
Overweight/obese	44	0.1 (−0.2, 0.4)	39.5 (37.4, 41.5)	21	0.0 (−0.5, 0.4)	38.9 (35.6, 42.2)
Serum 25-OH-vitamin D§						
≥50, nmol/L	121	0.1 (−0.1, 0.3)	29.4 (27.9, 30.9)	61	0.1 (−0.1, 0.3)	28.2 (26.0, 30.3)
≤50, nmol/L	75	−0.2 (−0.4, 0.0)	30.4 (28.1, 32.6)	45	0.0 (−0.3, 0.3)	30.1 (27.4, 32.8)
Vitamin D, daily intake¶						
Adequate >10 µg/day	55	0.0 (−0.2, 0.3)	30.3 (27.9, 32.7)	50	−0.1 (−0.3, 0.1)	28.0 (26.0, 30.0)
Inadequate ≤10 µg/day	143	0.0 (−0.2, 0.1)	29.5 (28.0, 31.0)	68	0.2 (0.0, 0.5)	29.6 (27.3, 31.9)
Ionised calcium**						
≥1.22 (mmol/L)	141	0.0 (−0.1,0.1)	30.0 (28.6, 31.4)	96	0.1 (0.0, 0.3)	29.3 (27.6, 31.0)
≤1.22 (mmol/L)	42	−0.1 (−0.4, 0.3)	28.1 (25.3, 31.0)	14	0.0 (−0.6, 0.6)	27.2 (21.6, 32.8)
Calcium, daily intake††						
4–10 years						
Adequate >800 mg/day	15	0.3 (−0.2, 0.8)	28.3 (25.7, 30.9)	9	0.4 (−0.4, 1.3)	27.9 (22.3, 33.6)
Inadequate ≤800 mg/day	22	0.3 (−0.1, 0.7)	31.7 (28.0, 35.3)	17	0.3 (−0.3, 0.8)	29.4 (25.7, 33.2)
11–18 years						
Adequate >1150 mg/day	51	−0.1 (−0.4, 0.2)	29.5 (26.9, 32.0)	22	0.0 (−0.3, 0.3)	27.9 (24.3, 31.6)
Inadequate ≤1150 mg/day	110	−0.1 (−0.2, 0.1)	29.6 (27.9, 31.4)	70	0.0 (−0.2, 0.2)	29.2 (27.0, 31.4)
Physical activity‡‡						
High activity	72	0.2 (0.0, 0.4)	27.0 (25.1, 29.0)	37	0.3 (0.0, 0.6)	27.3 (24.5, 30.2)
Intermediate activity	65	0.0 (−0.2, 0.2)	29.6 (27.5, 31.8)	41	0.1 (−0.2, 0.4)	30.3 (27.7, 32.9)
Low activity	45	−0.3 (−0.6, 0.0)	32.2 (29.7, 34.7)	23	−0.3 (−0.7, 0.1)	31.1* (27.3, 34.8)

BA-adj.: BA automatically measured by the software program BoneXpert, which assesses skeletal age from 21 bones in a digitalised X-ray of the left hand.

Z-score: The SD difference between the patient’s BMD measurement assessed with DXA, and the mean BMD of an age-matched and gender-matched reference.

* Total fat: Missing, 1.

† Parental education: Low level defined as education ≤13 years in both parents, Intermediate; One or both parents with university education up to 4 years, High; One or both parents with university education ≥5 years. JIA, missing 3, Controls, missing 39, total fat, missing 38.

‡ Iso-BMI according to the International Obesity Task Force recommendations corresponding to adult BMI (underweight: BMI≤18.5 kg/m2, overweight: BMI 25–29.9, obesity BMI≥30), overweight and obesity combined. JIA, missing 1.

§ Sampled once, at study visit 2, taken place throughout the year, August–June. JIA, missing 2, Controls, missing 12, Deficiency defined as ≤50, nmol/L.

¶ Estimates of daily intake (dietary and supplemental) based on data from FFQ. JIA, missing 15. Controls, missing 19. Inadequate intake ≤10 µg/day defined according to national recommendations.

** Sampled once, at study visit 2. Ionised calcium. JIA, missing 10. Controls, missing11. Deficiency defined as ≤1.22 nmol/L.

†† Estimates of daily intake (dietary and supplemental) based on data from self-reported FFQ. JIA, missing 15. Controls, missing 14. Inadequate intake is defined according to national recommendations.

‡‡ Physical activity question adopted from the WHO Health Behaviour in Schoolchildren: ‘Not during the average school day, how many days per week do you play sports or exercise to the point where you breathe heavily and/or sweat?’ The responses were divided into three categories. High activity: >4 times per week, Intermediate activity: 2–3 times per week, Low activity: 1 time per week or less. JIA, missing 16, Controls, missing 17.

BA, bone age; BMD, bone mineral density; BMD L1-L4, bone mineral density Lumbar spine 1-4; BMI, body mass index; BMI, age- and sex-adjusted body mass index; Ca, Calcium; DXA, dual-energy X-ray absorptiometry; FFQ, self-reported food frequency questionnaires; JIA, juvenile idiopathic arthritis; vit-D, vitamin D.

### BMD in relation to disease characteristics

BA-adjusted BMD L1-L4 Z-score was slightly lower in children with JIA and late disease onset, and in those with a long disease duration, compared with those who were younger at disease onset or had a shorter disease duration (table 6). Some minor differences were seen between the JIA categories. Children with polyarticular RF negative JIA had the lowest BA-adjusted BMD Z-score, −0.2 (95% CI −0.5, 0.1), compared with 0.2 (95% CI −0.1, 0.4) in children with oligoarticular persistent JIA. The number of children in the other categories was few, and the estimates uncertain. Children with JIA who had ever used bDMARDs or systemic steroids had lower BA-adjusted BMD Z-score of −0.2 (95% CI −0.4, 0.0) and −0.2 (95%CI −0.4, 0.1), respectively, compared with those who had never used DMARDs or systemic steroids 0.2 (95% CI −0.1, 0.4) and 0.1 (95% CI −0.1, 0.2) (table 6). Children with active disease had a slightly lower BA-adjusted BMD Z-score, −0.1 (95% CI −0.3, 0.1) compared with 0.2 (95% CI 0.0, 0.5) in those in remission off medication.

**Table 6:** BMD and total fat composition in JIA in relation to disease characteristics

Visit 2	N	BA-adj. BMD L1-L4, Z-scoren=198	Total fatn=198
		Mean (95% CI)	% (95% CI)
Age at disease onset, years			
0–4	83	0.1 (−0.1, 0.3)	29.1 (27.2, 31.0)
5–9	60	0.0 (−0.3, 0.2)	29.5 (27.1, 31.8)
10–16	55	−0.1 (−0.4, 0.1)	30.9 (28.4, 33.5)
Disease duration, years			
2–5	77	0.0 (−0.2, 0.2)	31.1 (29.1, 33.1)
6–8	43	0.1 (−0.2, 0.4)	29.6 (27.2, 32.1)
>8	78	−0.1 (−0.3, 0.1)	28.4 (26.3, 30.5)
JIA category*			
Oligoarticular persistent	61	0.2 (−0.1, 0.4)	29.8 (27.6, 32.1)
Oligoarticular extended	24	−0.1 (−0.5, 0.3)	30.4 (27.1, 33.7)
Polyarticular RF negative	45	−0.2 (−0.5, 0.1)	29.8 (27.4, 32.2)
Polyarticular RF positive	3	0.5 (−0.7, 1.8)	38.5 (36.5, 40.5)
Psoriatic arthritis	10	0.1 (−0.8, 0.9)	32.8 (26.5, 39.2)
Enthesitis-related arthritis	23	0.1 (−0.3, 0.4)	29.1 (24.0, 34.2)
Systemic arthritis	7	0.1 (−0.9, 1.1)	25.9 (20.5, 31.3)
Undifferentiated arthritis	25	−0.1 (−0.5, 0.3)	27.9 (23.7, 32.1)
DMARDs† ever			
No DMARDS	35	0.2 (−0.1, 0.4)	27.2 (24.0, 30.4)
csDMARDS only	63	0.2 (−0.1, 0.4)	29.1 (27.2, 31.1)
bDMARDS	100	−0.2 (−0.4, 0.0)	31.0 (29.1, 32.8)
Systemic steroids ever			
No systemic steroids	146	0.1 (−0.1, 0.2)	29.9 (28.5, 31.4)
Systemic steroids ever	52	−0.2 (−0.4, 0.1)	29.1 (26.7, 31.5)
Disease activity‡			
Remission off medication	40	0.2 (−0.1, 0.5)	26.1 (23.5, 28.8)
Inactive disease	89	0.0 (−0.2, 0.2)	30.1 (28.4, 31.8)
Active disease	69	−0.1 (−0.3, 0.1)	31.3 (28.9, 33.6)

BA-adj.: BA automatically measured by the software program BoneXpert, which assesses skeletal age from 21 bones in a digitalised X-ray of the left hand.

Z-score: the standard deviation difference between the patient’s BMD measurement assessed with Dual-energy X-ray absorptiometry (DXA), and the mean BMD of an age- and gender-matched reference population.

* JIA category according to The International League of Associations for Rheumatology classification criteria.

† csDMARDs include methotrexate, ciclosporin, mycophenolate mofetil, hydroxychloroquine. bDMARDs include etanercept, infliximab, adalimumab, tocilizumab, abatacept, rituximab, canakinumab.

‡ Disease status categorised according to Wallace *et al*^17^ and Wallace *et al*.^16^ Remission off medication=inactive disease for ≥12 months. Inactive disease=inactive disease off medication for less than 12 months, or inactive disease on medication independent of the duration of the inactive state. Active disease=flare or continuous active disease.

BA, bone age; bDMARDs, biological DMARDs; BMD, bone mineral density; BMD L1-L4, bone mineral density Lumbar spine 1-4; csDMARDs, conventional synthetic DMARDs; DMARDs, disease-modifying antirheumatic drugs; JIA, juvenile idiopathic arthritis; RF, rheumatoid factor.

### Body fat

No substantial differences in total fat percentage were seen between children with JIA and controls or in children with JIA between visits 1 and 2 (tables3  4). Factors that seemed to affect the fat percentage both in children with JIA and controls were low parenteral education, low serum vitamin D levels, and low physical activity. For all these factors, the mean fat percentage was >30% (table 5). The highest fat percentage among children with JIA was found in those with late disease onset 30.9% (95% CI 28.4%, 33.5%), short disease duration 31.1% (95% CI 29.1%, 33.1%), psoriatic arthritis JIA 32.8% (95% CI 26.5%, 39.2%), active disease 31.3% (95% CI 28.9%, 33.695% CI) and in those who had ever used bDMARDs 31.0% (95% CI 29.1%, 32.8%) (table 6).

## Discussion

In this Norwegian cohort study, we found no differences in BMD between children with JIA and controls; the BMD was within the normal reference range. Although children with JIA tended to have slightly lower BMD Z-scores in all measured locations compared with controls, the differences were minor and not statistically robust. Also, no substantial differences in BMD were seen between visit 1 and the 2-year follow-up among children with JIA. BMD Z-scores were slightly lower for children with JIA with low-educated parents, underweight, low serum vitamin D or ionised calcium levels. A more robust association was seen between physical activity levels and BMD both for children with JIA and controls, where high physical activity was associated with higher BMD Z-score. Late disease onset, long disease duration, polyarticular RF negative JIA, active disease and use of bDMARDS or systemic steroids were factors associated with a slightly lower BMD Z-score.

The strength of our study is the large study cohort, with JIA in all age groups from 4 to 16 years, with all JIA categories included, and an age-matched and sex-matched control cohort. Since the inclusion took place at three centres across the country, it provides representative data for children with JIA in Norway. Validated outcome measures were used for patient-reported and physician-reported outcomes. The clinical examination was performed by experienced paediatricians following a standardised study protocol. BMD Z-scores were adjusted for BA to reduce underestimation or overestimation of Z-scores that are based on the general population and not a patient group.

Some limitations must be mentioned. First, DXA in children is flawed with additional biases, such as variations between different DXA systems and differences between normative paediatric reference databases.^24^ The extensive study programme may explain the younger median age of children who declined participation, suggesting that selection bias cannot be ruled out. Another limitation is the potential selection of more children with active disease within the study group. In some JIA categories, particularly those with the most severe subtypes, such as systemic arthritis and RF positive polyarticular JIA, and also in the underweight group, the low number of participants made comparisons difficult due to low statistical power. An additional limitation is that the physical activity questions, which were adopted from the WHO’s HBSC study, do not provide specific details about the types of activities performed by the children, such as weight-bearing exercises, which are known to be important for bone mineralisation.^25^ No cross-calibration equations were done between the DXA devices at the three different study centres to ensure comparability of data on different scanners. However, there was little variation in BMD Z-score between centres (results not shown). An additional limitation arises from the use of the Bland-Altman plot to compare individual BHI-SDS values with BA-adjusted BMD L1-L4 scores. This plot cannot be considered a definitive method for evaluating the usability of BHI-SDS against DXA, nor can it serve as conclusive validation of comparability between the two methods. Further analyses are therefore needed.^26^ However, it does provide valuable insights into BHI-SDS in a clinical cohort of children with JIA and controls. As there were few differences when comparing JIA with controls regarding BMD and sociodemographic factors, we omitted additional statistical analysis.

Our results differ from several other studies. A systematic review of 100 papers on fractures and BMD in children with JIA from 2016 concluded that most studies show that children with JIA have reduced BMD independent of the glucocorticoid effect. The reduction was in the range of −0.5 to −1.5 in Z-scores. Systemic arthritis and polyarticular arthritis were associated with larger reductions than oligoarticular arthritis.^27^ Decreased total body BMD (absolute values and Z-scores) in children with JIA compared with controls was also found in a Norwegian study from 2018.^28^ In contrast, a Swedish study from 2012, aiming to study BMD in a cohort of children and adolescents with JIA before and after a physical exercise programme, concluded that the included cohort of 54 participants had a normal BMD compared with a healthy age-matched and sex-matched reference group at baseline.^29^ Similar results were found in a study from Thailand where only 5.3% of the participants had low total body BMD Z-score (defined as a BMD Z-score <−2 SD), and none had low LS BMD Z-score.^30^

One possible explanation for the normal BMD Z-scores observed in our study is that children and adolescents with JIA have access to better treatment options from the time of diagnosis, compared with older studies. Systemic glucocorticoids are now typically administered for the shortest possible duration and may have been given to manage arthritis, flares or uveitis. In our study, 50% of children with JIA had received treatment with bDMARDS at some point. Unlike many other countries, Norway offers equal treatment options to everyone, regardless of insurance or socioeconomic status. In general, therapeutic approaches vary between countries. Similarly, national recommendations on lifestyle advice, including physical activity guidelines, activity restrictions, diet and vitamin supplementation, differ as well. Comparison between studies may also be hampered due to different study groups, different degree of selection and disease severity.

Many sociodemographic factors have been associated with low BMD in the general population. In contrast to our study, a Mexican study from 2022 found that the BMD in obese children was higher than in normal-weight children, possibly due to their increased lean mass.^31^ This conclusion aligns with a systematic review from 2017.^32^ Like the tendency seen worldwide and in line with WHO concerns, both children with JIA and controls in our study had a relatively high fat percentage.^33^ A higher body fat percentage in children with JIA compared with controls has previously been reported.^34^ In accordance with our results, it is well known that underweight is a risk factor for reduced BMD.^35^

Although we found only weak associations between vitamin D, ionised calcium serum levels, inadequate intake and BMD, other studies have demonstrated more pronounced associations.^30 36 37^ A study from 2023 demonstrated a positive relationship between serum vitamin D levels and total body BMD Z-scores.^30^ An Italian study from 2014 found that children with JIA and vitamin D deficiency had lower BMD than those with normal serum vitamin D levels. In contrast to our results, they also found that children with JIA had significantly reduced vitamin D levels compared with controls.^36^ In an Indian study, low bone mass was associated with decreased dietary intake of vitamin D and calcium.^37^ The minor associations between vitamin D and BMD levels observed between JIA and controls in our study may be explained by the high prevalence of children in Norway taking cod liver oil or other vitamin D supplements.^38^

The striking association between BMD and levels of physical activity found in our study was not unexpected. However, it was both surprising and reassuring to find that children with JIA were as physically active as children in the control group. In contrast, related studies have reported that children with JIA are less physically active than controls.^39 40^ In a Tunisian study from 2020, children and adolescents with JIA were less physically active than recommended from a general health perspective and less physically active than their age-matched and sex-matched controls.^41^ Physical activity restrictions were traditionally recommended for children with JIA and other rheumatic diseases. In contrast, there is now increasing evidence that physical activity can reduce pain, fatigue, muscle weakness and improve bone health.^42 43^ Studies also demonstrate that the benefit of physical activity clearly outweighs the risks.^44^ It has also been shown that specific exercise programmes may increase the BMD in children with JIA.^29^

Several studies have examined BMD in children with JIA in association with disease severity and disease duration.^45^,^47^ In accordance with our study, two Egyptian studies from 2023 and 2020 and a Chinese study from 2015 found a correlation between BMD Z-score and disease duration.^45^,^47^ In contrast to these results, disease duration was not found to have a significant correlation with BMD in an Indian study from 2014, but they found a positive correlation between BMD and age at disease onset.^37^ This was also found in the study from China from 2015.^47^ Similar to our study, several studies have found that children with polyarticular JIA had lower BMD compared with other categories.^45^,^47^ It is well known, and confirmed by many studies and also seen as trends in our study, that use of systemic glucocorticoids is associated with decreased BMD,^12 37^ and that active disease correlates with lower BMD.^37 46 47^ In an Egyptian study from 2023, there was no difference in BMD between those who received biological treatment vs those who did not,^12^ in contrast to our study where we found a slightly lower BMD Z-score in children with JIA who had ever used bDMARDs.

As a chronic inflammatory disease with onset during childhood, JIA can lead to disrupted bone metabolism and eventually reduced BMD. Early treatment with DMARDs, including bDMARDs, has improved the overall outcome for children with JIA and reduced the requirement for glucocorticoids, though long-lasting low-grade inflammation may still impact bone health. Physical activity is essential for bone and muscle development, particularly in children with JIA, as it helps counteract the negative effects of inflammation during a critical period for bone mass accrual. Despite minimal BMD differences between children with JIA and controls, the results of this study support that timely and effective treatment of JIA, along with proper management of disease activity together with encouragement of physical activity, can improve bone health and lower the risk of osteoporosis.

In the future, microscan^48^ can offer a detailed and powerful method for assessing bone quality and microarchitecture beyond traditional BMD measurements and radiogrammetry. It may be a valuable tool for evaluating bone structure and strength in children with JIA. However, its use requires careful consideration of developmental factors, radiation exposure and the availability of reference data.

## Conclusions

In this study on bone health, we found that there were no substantial differences in BMD Z-scores in JIA compared with controls, but we found a pattern of decreasing BMD Z-scores with decreasing physical activity levels. Our results underline the importance of early disease control, use of steroid-sparing medications, and physical activity to optimise bone health in children with JIA.

## Supplementary material

10.1136/rmdopen-2025-005605 online supplemental file 1

## Data Availability

Data are available on reasonable request.

## References

[1] Petty RE, Southwood TR, Manners P (2001). International League of Associations for Rheumatology classification of juvenile idiopathic arthritis: second revision. J Rheumatol.

[2] Thierry S, Fautrel B, Lemelle I (2014). Prevalence and incidence of juvenile idiopathic arthritis: a systematic review. Joint Bone Spine.

[3] Hestetun SV, Rudsari HK, Jaholkowski P (2025). Incidence and Genetic Risk of Juvenile Idiopathic Arthritis in Norway by Latitude. Arthritis Rheumatol.

[4] d’Angelo DM, Di Donato G, Breda L (2021). Growth and puberty in children with juvenile idiopathic arthritis. Pediatr Rheumatol Online J.

[5] Alwis G, Rosengren B, Stenevi-Lundgren S (2010). Normative dual energy X-ray absorptiometry data in Swedish children and adolescents. Acta Paediatr.

[6] Di Marcello F, Di Donato G, d’Angelo DM (2022). Bone Health in Children with Rheumatic Disorders: Focus on Molecular Mechanisms, Diagnosis, and Management. *IJMS*.

[7] Chevalley T, Rizzoli R (2022). Acquisition of peak bone mass. Best Pract Res Clin Endocrinol Metab.

[8] Crabtree NJ, Arabi A, Bachrach LK (2014). Dual-energy X-ray absorptiometry interpretation and reporting in children and adolescents: the revised 2013 ISCD Pediatric Official Positions. J Clin Densitom.

[9] Shalof H, Dimitri P, Shuweihdi F (2021). Which skeletal imaging modality is best for assessing bone health in children and young adults compared to DXA? A systematic review and meta-analysis. Bone.

[10] Weber DR, Boyce A, Gordon C (2019). The Utility of DXA Assessment at the Forearm, Proximal Femur, and Lateral Distal Femur, and Vertebral Fracture Assessment in the Pediatric Population: 2019 ISCD Official Position. J Clin Densitom.

[11] Vasil E, M Nesbitt C, Toomey C (2024). Bone health and physical activity in adolescents with juvenile idiopathic arthritis: a cross-sectional case-control study. Pediatr Rheumatol Online J.

[12] Eid R, Abdelsalam M, Fathy AA (2023). Bone mineral density in egyptian children with juvenile idiopathic arthritis: possible correlation to serum RANKL / osteoprotegerin (OPG) ratio and OPG gene polymorphisms. Pediatr Rheumatol Online J.

[13] Júlíusson PB, Hjelmesæth J, Bjerknes R (2017). New curves for body mass index among children and adolescents. Tidsskr Nor Laegeforen.

[14] King A, Wold B, Tudor-Smith C (1996). The health of youth. A cross-national survey. WHO Reg Publ Eur Ser.

[15] Jahre H, Grotle M, Småstuen M (2021). Risk factors and risk profiles for neck pain in young adults: Prospective analyses from adolescence to young adulthood-The North-Trøndelag Health Study. PLoS One.

[16] Wallace CA, Giannini EH, Huang B (2011). American College of Rheumatology provisional criteria for defining clinical inactive disease in select categories of juvenile idiopathic arthritis. Arthritis Care Res (Hoboken).

[17] Wallace CA, Ruperto N, Giannini E (2004). Preliminary criteria for clinical remission for select categories of juvenile idiopathic arthritis. J Rheumatol.

[18] Consolaro A, Giancane G, Schiappapietra B (2016). Clinical outcome measures in juvenile idiopathic arthritis. *Pediatr Rheumatol Online J*.

[19] Filocamo G, Davì S, Pistorio A (2010). Evaluation of 21-numbered circle and 10-centimeter horizontal line visual analog scales for physician and parent subjective ratings in juvenile idiopathic arthritis. J Rheumatol.

[20] Selvaag AM, Ruperto N, Asplin L (2001). The Norwegian version of the Childhood Health Assessment Questionnaire (CHAQ) and the Child Health Questionnaire (CHQ). Clin Exp Rheumatol.

[21] Annexstad EJ, Bollerslev J, Westvik J (2019). The role of delayed bone age in the evaluation of stature and bone health in glucocorticoid treated patients with Duchenne muscular dystrophy. Int J Pediatr Endocrinol.

[22] Martin DD, Calder AD, Ranke MB (2022). Accuracy and self-validation of automated bone age determination. Sci Rep.

[23] Thodberg HH, van Rijn RR, Tanaka T (2010). A paediatric bone index derived by automated radiogrammetry. Osteoporos Int.

[24] Ma J, Siminoski K, Alos N (2015). The choice of normative pediatric reference database changes spine bone mineral density Z-scores but not the relationship between bone mineral density and prevalent vertebral fractures. J Clin Endocrinol Metab.

[25] Mesquita E de L, Exupério IN, Agostinete RR (2022). The Combined Relationship of Vitamin D and Weight-Bearing Sports Participation on Areal Bone Density and Geometry Among Adolescents: ABCD - Growth Study. J Clin Densitom.

[26] Augdal T, Angenete O, Zadig P (2024). The assessment of bone health in children with juvenile idiopathic arthritis; comparison of different imaging-based methods. Pediatr Rheumatol Online J.

[27] Huber AM, Ward LM (2016). The impact of underlying disease on fracture risk and bone mineral density in children with rheumatic disorders: A review of current literature. Semin Arthritis Rheum.

[28] Risum K, Edvardsen E, Godang K (2019). Physical Fitness in Patients With Oligoarticular and Polyarticular Juvenile Idiopathic Arthritis Diagnosed in the Era of Biologics: A Controlled Cross-Sectional Study. *Arthritis Care Res (Hoboken*).

[29] Sandstedt E, Fasth A, Fors H (2012). Bone health in children and adolescents with juvenile idiopathic arthritis and the influence of short-term physical exercise. Pediatr Phys Ther.

[30] Charuvanij S, Malakorn H, Densupsoontorn N (2023). Bone Mineral Density and Serum 25OHD in Children and Adolescents With Juvenile Idiopathic Arthritis. Clin Pediatr (Phila).

[31] López-Peralta S, Romero-Velarde E, Vásquez-Garibay EM (2022). Bone mineral density and body composition in normal weight, overweight and obese children. BMC Pediatr.

[32] van Leeuwen J, Koes BW, Paulis WD (2017). Differences in bone mineral density between normal-weight children and children with overweight and obesity: a systematic review and meta-analysis. Obes Rev.

[33] Umekar S, Joshi A (2024). Obesity and Preventive Intervention Among Children: A Narrative Review. Cureus.

[34] Lien G, Selvaag AM, Flatø B (2005). A two-year prospective controlled study of bone mass and bone turnover in children with early juvenile idiopathic arthritis. Arthritis Rheum.

[35] Bialo SR, Gordon CM (2014). Underweight, Overweight, and Pediatric Bone Fragility: Impact and Management. Curr Osteoporos Rep.

[36] Stagi S, Bertini F, Cavalli L (2014). Determinants of vitamin D levels in children, adolescents, and young adults with juvenile idiopathic arthritis. J Rheumatol.

[37] Dey S, Jahan A, Yadav TP (2014). Measurement of bone mineral density by dual energy X-ray absorptiometry in juvenile idiopathic arthritis. Indian J Pediatr.

[38] Cetrelli L, Bletsa A, Lundestad A (2022). Vitamin D, oral health, and disease characteristics in juvenile idiopathic arthritis: a multicenter cross-sectional study. BMC Oral Health.

[39] Milatz F, Pedersen MJ, Klotsche J (2024). Physical (in)activity and screen-based media use of adolescents with juvenile idiopathic arthritis over time - data from a German inception cohort. Pediatr Rheumatol Online J.

[40] Bos GJFJ, Lelieveld OTHM, Armbrust W (2016). Physical activity in children with Juvenile Idiopathic Arthritis compared to controls. Pediatr Rheumatol Online J.

[41] Fazaa A, Sellami M, Ouenniche K (2021). Physical activity assessment in children and adolescents with juvenile idiopathic arthritis compared with controls. Arch Pediatr.

[42] Liu WY, Li HM, Jiang H (2024). Effect of exercise training on heath, quality of life, exercise capacity in juvenile idiopathic arthritis: a meta-analysis of randomized controlled trials. Pediatr Rheumatol Online J.

[43] Dawoud A, Blitz J, Moonaz S (2024). Feasibility and Acceptability of Yoga for Adolescents with Juvenile Idiopathic Arthritis. Children (Basel).

[44] Gualano B, Bonfa E, Pereira RMR (2017). Physical activity for paediatric rheumatic diseases: standing up against old paradigms. Nat Rev Rheumatol.

[45] Hassan A-EA, Rayan MMM, Abdel-Aziz TM (2020). Value of Screening for Osteoporosis among Children with Juvenile Idiopathic Arthritis. EJHM.

[46] Soliman SG, Nofal DA, Labeeb AA (2023). Assessment of bone mineral density and bone turnover markers in patients with juvenile idiopathic arthritisy. Voprosy Gematologii/Onkologii i Immunopatologii v Pediatrii.

[47] Tang T, Tang X, Xu L (2015). Evaluation of bone mass in children and young adults with juvenile idiopathic arthritis. Clin Exp Rheumatol.

[48] Gabel L, Kent K, Hosseinitabatabaei S (2023). Recommendations for High-resolution Peripheral Quantitative Computed Tomography Assessment of Bone Density, Microarchitecture, and Strength in Pediatric Populations. Curr Osteoporos Rep.

