# Supplementary material for: Bone health in juvenile idiopathic arthritis compared with controls based on a Norwegian observational study
Source: RMD Open. 2025 Jun 19;11(2):e005605. doi: 10.1136/rmdopen-2025-005605 (PMC12182186; doi:10.1136/rmdopen-2025-005605)
Supplement: online supplemental file 1 [file rmdopen-11-2-s001.docx]

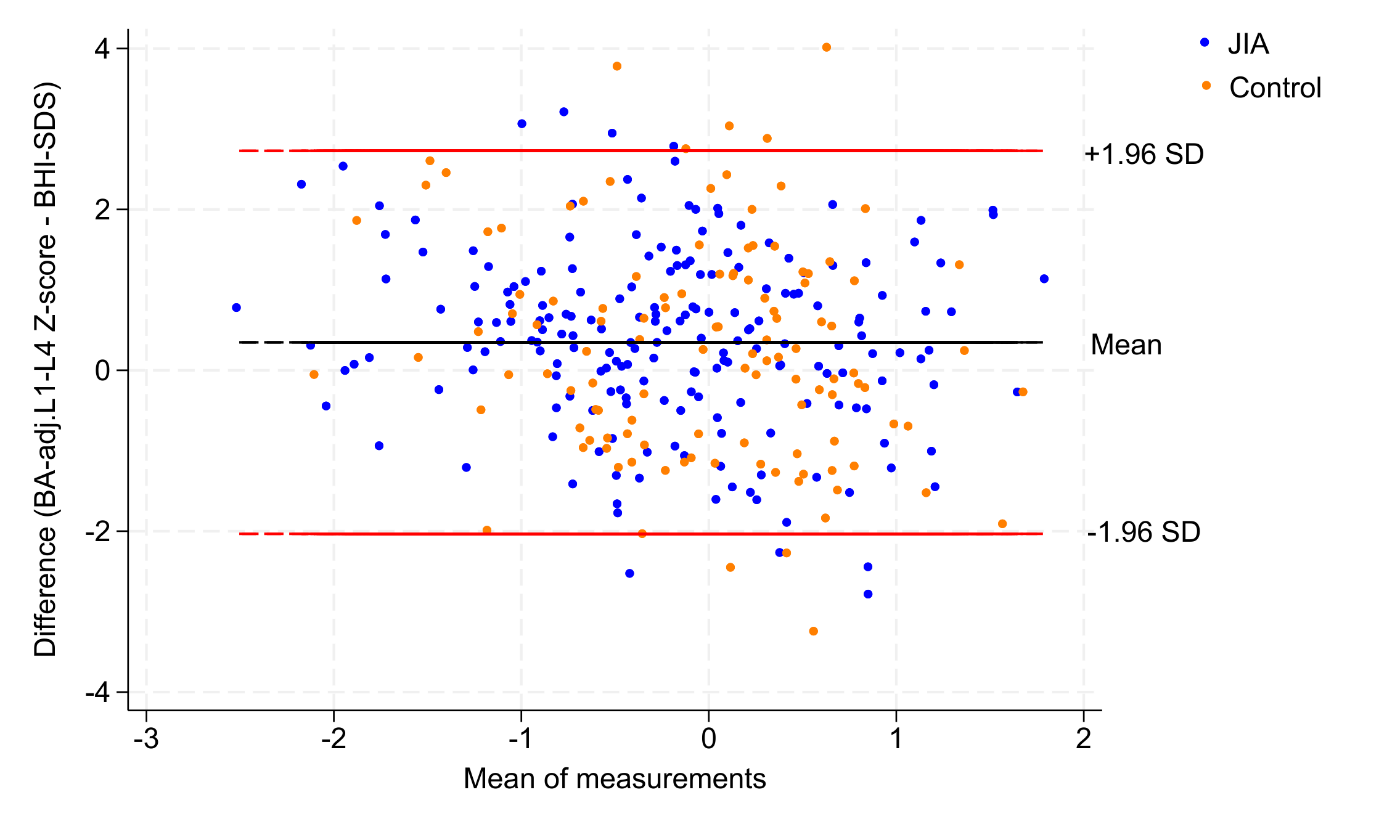


**Supplemental Figure 1.** Comparison of individual bone health index standard deviation score (BHI-SDS) values with bone age-adjusted bone mineral density L1-L4 (BA-adjusted BMD L1-L4) Z-scores in children with juvenile idiopathic arthritis (JIA) (blue dots) and controls (orange dots) at study visit 2. The X-axis of the Bland- Altman plot represents the mean of the measurements, while the Y-axis shows the difference between BA-adj. L1-L4 Z-score and BHI-SDS values. The black horizontal line indicates the mean difference, and the red lines represent the 95%-limits of agreement (±1.96 standard deviations). Bone age was automatically measured by the software program BoneXpert, which assesses skeletal age from 21 bones in a digitalized radiograph of the left hand and used for adjustments of BMD Z-scores. BHI-SDS is derived from the same radiograph using automated measurements of metacarpal length, width and cortical thickness.
